# Rapid generation of clinical-grade antiviral T cells: selection of suitable T-cell donors and GMP-compliant manufacturing of antiviral T cells

**DOI:** 10.1186/s12967-014-0336-5

**Published:** 2014-12-16

**Authors:** Sabine Tischer, Christoph Priesner, Hans-Gert Heuft, Lilia Goudeva, Wolfgang Mende, Marc Barthold, Stephan Kloeß, Lubomir Arseniev, Krasimira Aleksandrova, Britta Maecker-Kolhoff, Rainer Blasczyk, Ulrike Koehl, Britta Eiz-Vesper

**Affiliations:** Institute for Transfusion Medicine, Hannover Medical School, Carl-Neuberg-Strasse 1, 30625 Hannover, Germany; Integrated Research and Treatment Center (IFB-Tx), Hannover Medical School, Carl-Neuberg-Strasse 1, 30625 Hannover, Germany; Institute of Cellular Therapeutics, Hannover Medical School, Feodor-Lynen Strasse 21, 30625 Hannover, Germany; Staff office for Quality Management in Clinical Research, Hannover Medical School, Feodor-Lynen Strasse 21, 30625 Hannover, Germany; Department of Paediatric Haematology and Oncology, Hannover Medical School, Carl-Neuberg-Strasse 1, 30625 Hannover, Germany

**Keywords:** Adoptive immunotherapy, Antiviral T cells, *alloCELL*, GMP-compliant manufacturing, CliniMACS CCS, Stem cell transplantation, Adoptive T-cell transfer

## Abstract

**Background:**

The adoptive transfer of allogeneic antiviral T lymphocytes derived from seropositive donors can safely and effectively reduce or prevent the clinical manifestation of viral infections or reactivations in immunocompromised recipients after hematopoietic stem cell (HSCT) or solid organ transplantation (SOT). Allogeneic third party T-cell donors offer an alternative option for patients receiving an allogeneic cord blood transplant or a transplant from a virus-seronegative donor and since donor blood is generally not available for solid organ recipients. Therefore we established a registry of potential third-party T-cell donors (allogeneic cell registry, *alloCELL*) providing detailed data on the assessment of a specific individual memory T-cell repertoire in response to antigens of cytomegalovirus (CMV), Epstein-Barr virus (EBV), adenovirus (ADV), and human herpesvirus (HHV) 6.

**Methods:**

To obtain a manufacturing license according to the German Medicinal Products Act, the enrichment of clinical-grade CMV-specific T cells from three healthy CMV-seropositive donors was performed aseptically under GMP conditions using the CliniMACS cytokine capture system (CCS) after restimulation with an overlapping peptide pool of the immunodominant CMVpp65 antigen. Potential T-cell donors were selected from *alloCELL* and defined as eligible for clinical-grade antiviral T-cell generation if the peripheral fraction of IFN-γ^+^ T cells exceeded 0.03% of CD3^+^ lymphocytes as determined by IFN-γ cytokine secretion assay.

**Results:**

Starting with low concentration of IFN-γ^+^ T cells (0.07-1.11%) we achieved 81.2%, 19.2%, and 63.1% IFN-γ^+^CD3^+^ T cells (1.42 × 10^6^, 0.05 × 10^6^, and 1.15 × 10^6^) after enrichment. Using the CMVpp65 peptide pool for restimulation resulted in the activation of more CMV-specific CD8^+^ than CD4^+^ memory T cells, both of which were effectively enriched to a total of 81.0% CD8^+^IFN-γ^+^ and 38.4% CD4^+^IFN-γ^+^ T cells. In addition to T cells and NKT cells, all preparations contained acceptably low percentages of contaminating B cells, granulocytes, monocytes, and NK cells. The enriched T-cell products were stable over 72 h with respect to viability and ratio of T lymphocytes.

**Conclusions:**

The generation of antiviral CD4^+^ and CD8^+^ T cells by CliniMACS CCS can be extended to a broad spectrum of common pathogen-derived peptide pools in single or multiple applications to facilitate and enhance the efficacy of adoptive T-cell immunotherapy.

**Electronic supplementary material:**

The online version of this article (doi:10.1186/s12967-014-0336-5) contains supplementary material, which is available to authorized users.

## Background

Infection or reactivation with cytomegalovirus (CMV), Epstein-Barr virus (EBV), adenovirus (ADV), and human herpesvirus (HHV) 6 are the most common causes of viral morbidity and mortality after hematopoietic stem cell transplantation (HSCT) or solid organ transplantation (SOT) [1-9]. The lack or low frequency of antiviral T cells and the delay in virus-specific T-cell reconstitution are critical factors in virally infected post-transplant patients. Functionally active antiviral T cells are crucial for the effective elimination and control of those life-threatening viral infections or reactivations [10-12]. Treatment with donor lymphocyte infusions (DLI) routinely separated from the seropositive stem cell donor can improve the clinical outcome of viral infection and leukaemia relapse, but it is (i) associated with a high risk of inducing graft-versus-host disease (GvHD), (ii) attended with impaired functionality of antiviral memory T cells in granulocyte colony-stimulating factor- (G-CSF-) mobilized stem cell donors [13-15], (iii) not suitable in high risk patients with seronegative donors and (iv) not available for patients receiving cord blood in HSCT or cadaveric transplants in SOT. Recent studies have shown that the adoptive transfer of T cells with selected antigen-specificities is an effective and safe treatment option for enhancing the long-term protection of patient immunity after engraftment and immune reconstitution without increasing the risk of GvHD [2-6,8,16-18]. The efficient treatment of high risk patients with seronegative donors requires the rapid recruitment of a suitable seropositive T-cell donor as well as an established and robust protocol for the timely manufacturing of antiviral T cells without long-term *ex vivo* stimulation. One promising option for providing potential T-cell donor is the allogeneic cell registry (*alloCELL*, http://www.alloCELL.org), which was established at Hannover Medical School within the last three years. The registry compiles screening results on the specific memory T-cell repertoire of potential donors in response to CMV, EBV, and ADV [19] and is now extended to polyoma virus (BK) and HHV6 [9] and thus will accelerate the adoptive T-cell therapy. Currently the enrichment of clinical-grade antigen-specific T cells from peripheral blood without long-term *ex vivo* manipulation can be performed by two major principles: the interferon-gamma (IFN-γ) based CliniMACS cytokine capture system (CCS) and the reversible peptide-MHC (pMHC) class I multimer technology. Both techniques are already successfully used for the selection of antiviral T cells in clinical settings [1-3,6-8,17,20,21]. The CliniMACS CCS method has the advantage that instead of single HLA-restricted peptides, recombinant proteins and overlapping peptide pools not subjected to HLA restriction can be used. These antigens enable the generation of a broad repertoire of both CD8^+^ cytotoxic T cells (CTLs) and CD4^+^ T helper (Th) cells specific to multiple epitopes [22]. Synthetic peptide pools covering the entire sequence of a pathogen protein are most suitable for manufacturing clinical-grade specific CD4^+^ and CD8^+^ T cells because they can be produced and controlled more easily than recombinant proteins under Good Manufacturing Practice (GMP) conditions [23].

**Figure 1:**
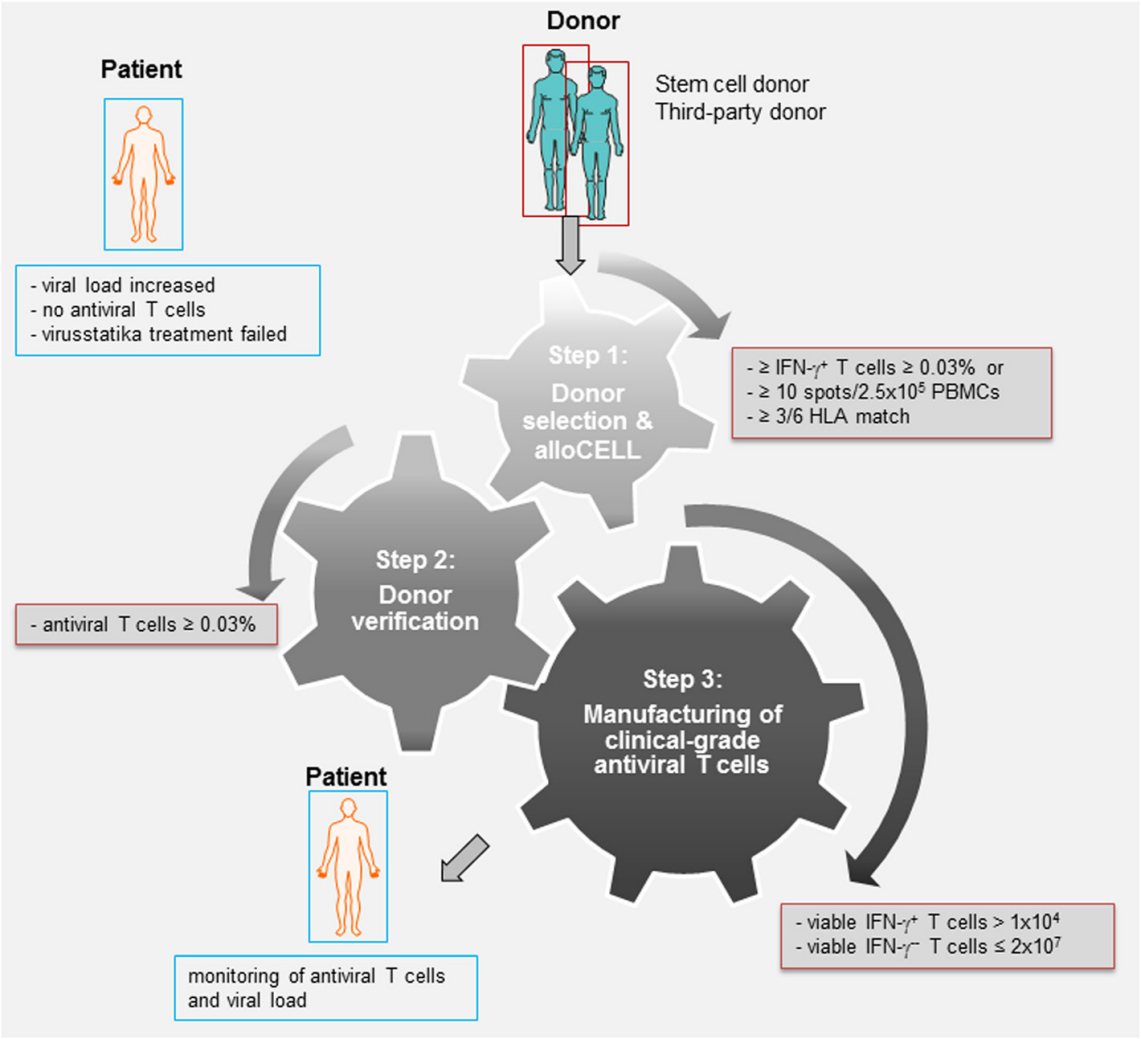
**Protocol for the rapid manufacture of clinical-grade antigen-specific T cells. **A three-step protocol for the rapid generation of clinical-grade antiviral T cells was established to facilitate the manufacture of specific T cells for adoptive transfer in pre-monitored patients. First Step: Selection of potential T-cell donors from the *alloCELL* registry (HLA type, virus serology and virus-specific T-cell response). Second Step: Verification of the donor’s specific T-cell frequencies (donor from *alloCELL*, stem cell or family donor) and prediction of the donor’s T-cell enrichment efficiency by small-scale MiniMACS CSA. A T-cell donor is classified as eligible if (a) the peripheral frequency of virus-specific IFN-γ^+^ T cells ≥0.03% of total CD3^+^ T cells and (b) the restimulation efficiency is twice as much as the unstimulated control. Third Step: Manufacturing of clinical-grade antiviral T cells by large-scale CliniMACS CCS. A CliniMACS CCS-enriched T-cell fraction (TCF) is classified as eligible if (a) number of viable IFN-γ^+^ T cells >1 × 10^4^ and (b) the number of viable IFN-γ^−^ T cells ≤2 × 10^7^.

To obtain a manufacturing license according to the German Medicinal Products Act (AMG) we first established a reproducible protocol for the rapid manufacture of clinical-grade T cells specific for CMV (Figure 1). Our results suggest that sufficient numbers of functionally active CMV-specific CD4^+^ and CD8^+^ T cells can be activated by using the overlapping peptide pool of the immunodominant CMV phosphoprotein 65 (pp65) as the stimulating agent and efficiently enriched by CliniMACS CCS with an adequate purity for adoptive T-cell transfer.

## Methods

### Allogeneic cell registry, *alloCELL*

Suitable third-party T-cell donors were selected from the allogeneic cell registry *alloCELL* (http://www.alloCELL.org) established at Hannover Medical School (MHH) as described previously [19]. Informed consent was obtained from all donors as approved by the Ethics Committee of Hannover Medical School. All donors belong to the active thrombocyte and blood donor pool of MHH’s Institute for Transfusion Medicine and were typed for HLA class I and class II alleles at the four-digit level by sequence-based typing [24]. The ever-expanding *alloCELL* registry documents specific so far T-cell frequencies against different epitopes of CMV, EBV, ADV, and HHV6 for 450 out of 1150 donors, best T-cell detection method, and results of functional and alloreactivity assays. Donors are classified as high, low, and non-responders according to the specific antiviral memory T-cell frequencies as described by Sukdolak *et al.* [19].

### Selection of a suitable CMV-specific T-cell donor

Three healthy donors with no acute infection and who were determined to be eligible by national standards for the donation of allogeneic blood products were selected from *alloCELL* as potential candidates for T-cell donation. Selection was performed at first on the basis of the CMV serostatus and the presence of CMV-specific T cells as monitored by IFN-γ EliSpot assay in response to the CMVpp65 overlapping peptide pool (CMVpp65_pp_) and pMHC pentamer staining if the donor was HLA-A*02:01-positive [13,19]. IFN-γ EliSpot assay was performed with 2.5 × 10^5^ peripheral blood mononuclear cells (PBMCs)/well using 1 μg/ml per peptide of CMVpp65_pp_ (Miltenyi Biotec, Bergisch Gladbach, Germany) for restimulation as described previously [19,25]. For a positive response 10 spots per well (spw)/2.5 × 10^5^ PBMCs were defined as cut-off. In addition, for HLA-A*02:01-positive donors peptide-specific CD8^+^ T cells were detected by pMHC pentamer staining (Proimmune, Oxford, UK; CMVpp65_495–503,_ epitope NLVPMVATV, shortened A02pp65_M_) as described in further studies [13,19].

To finally define these donors as suitable for clinical-grade antiviral T-cell generation a detailed analysis of antiviral T-cell frequencies was performed by cytokine secretion assay (CSA). For recruitment, the starting frequency of IFN-γ^+^ T cells had to exceed 0.03 of CD3^+^ lymphocytes and >2× the negative control value (cut-off for positive response).

### Detection of IFN-γ secreting CMV-specific T cells by cytokine secretion assay

The non-GMP IFN-γ MiniMACS CSA (IFN-γ Secretion Assay – Cell Enrichment and Detection Kit, Miltenyi Biotec) was performed according to the manufacturer’s instructions and was used: (1) to verify the starting frequency of the donor’s CMV-specific memory T-cells, (2) to predict the T-cell enrichment efficiency, and (3) as a control in parallel to the clinical-scale CliniMACS CCS enrichment procedure. By this the acceptability of the starting leukapheresis material and non-specific spontaneous release of IFN-γ in the unstimulated negative control was determined. PBMCs were cultured *ex vivo* for four hours in T-CM alone (negative control), with 1 μg/ml per peptide of the CMVpp65_pp_, and with 2 μg/ml staphylococcal enterotoxin B (positive control; SEB, Sigma-Aldrich, Hamburg, Germany), respectively. IFN-γ^+^ CMVpp65-specific T cells were specifically captured during the magnetic cell sorting (MACS) enrichment processes by anti-IFN-γ-phycoerythrin (PE) antibody and paramagnetic anti-PE mircobeads. The relevant MiniMACS CSA cell fractions were used for a detailed analysis of IFN-γ^+^ T-cell subsets. The distribution of viable and dead cells in these fractions was analysed by 7AAD (7-amino-actinomycin D) staining (BD Biosciences, Heidelberg, Germany). The percentage of viable IFN-γ^+^ cells was further analysed by staining the cells with anti-CD45-allophycocyanin with cyanin-7 (APC/Cy7), anti-CD56-phycoerythrin with cyanin-7 (PE/Cy7), and anti-CD3-fluorescein isothiocyanate (FITC, all BD Biosciences) mAbs. At least 10,000 events were acquired in the viable CD45^+^ leukocyte gate for each analysis (FACSCantoII, BD Biosciences). CD3^+^IFN-γ^+^, CD8^+^IFN-γ^+^ and CD4^+^IFN-γ^+^ T-cell populations were gated based on the scatter properties of viable 7AAD^−^CD45^+^CD56^−^CD3^+^ T lymphocytes.

### Generation of clinical-grade CMV-specific T cells by CliniMACS CCS

CMVpp65-specific T cells were enriched under GMP conditions by using the IFN-γ CliniMACS Cytokine Capture System (CliniMACS CCS) with the specified components and reagents according to the manufacturer’s instructions (Miltenyi Biotec).

After washing the donor’s leukapheresis with CliniMACS PBS/EDTA buffer, leukocytes were adjusted to a total of 2 × 10^9^ leukocytes in 200 ml GMP compatible serum-free TexMACS medium and stored overnight in 500 ml MACS GMP Cell Differentiation Bags at 37°C and 5% CO_2_. Next morning, a total of 1 × 10^9^ leukocytes were used for *ex vivo* stimulation with the GMP-grade CMVpp65 peptide pool (CMVpp65_pp_, MACS GMP PepTivator HCMV pp65, 1 μg/ml per peptide) for four hours at 37°C. For control MiniMACS CSA was performed in parallel with 1 × 10^7^ leukocytes from the overnight incubation with the CMVpp65_pp_. Enrichment of IFN-γ secreting leukocytes during CliniMACS CCS was performed by immunomagnetic separation using the CliniMACS Tubing Set and the CliniMACS Cytokine Capture System, which consisted of the CliniMACS Catchmatrix Reagent and the CliniMACS IFN-γ Enrichment Reagent. CliniMACS PBS/EDTA buffer was supplemented with 0.5% HSA (human serum albumin) and used for all washing steps and the elution of the final T-cell product. For cryopreservation the eluate fraction was adjusted to 2.86% HSA, 7.5% DMSO (dimethyl sulfoxide), aliquoted, subsequently processed in a controlled-rate freezer, and finally transferred to −140°C or lower in the vapour phase above liquid nitrogen for long-term storage. A fully automated microbial detection system was used for microbiological testing (sterility) of the leukapheresis and the CliniMACS CCS T-cell fraction. Total cell number and total viability of the final T-cell product was determined by light microscopy using trypan blue. Due to the low cell numbers in the final T-cell product, cell counting by light microscopy was performed by two staff members with each counting 16 large scares. For all other process-attendant cell fractions total cell number was counted by the full-automated Hemocounter (Coulter ACTdiff, Beckman Coulter), while total viability was analysed by flow cytometry (BD FACSCantoII) using 7AAD.

### Quality control: assessment of the final T-cell product by flow cytometry

Specific risk-based acceptance criteria were defined as GMP-compliant prerequisites apart from the GMP-compliant controlled manufacturing and testing environment (Additional file 1: Table S1). Criteria were defined in consideration of published pre-clinical studies, statistical inference, and the long-lasting expertise with already established protocols for the generation of clinical-grade cell products using CliniMACS technologies (e.g. selection of CD34^+^ cells). Samples of the following fractions from CliniMACS CCS and MiniMACS CSA processes were collected and analysed: leukapheresis, original fraction (OF, after restimulation and before magnetic enrichment), T-cell fraction (TCF, after magnetic enrichment), waste fraction (WF, washing effluent) and negative fraction (NF, cells not retained on the column). Additionally the stability of the final product was assessed in reference samples stored for a total of 72 hours after leukapheresis and analysed after 48 (stabi48), 54 (stabi54) and 72 (stabi72) hours (h).

**Figure 2:**
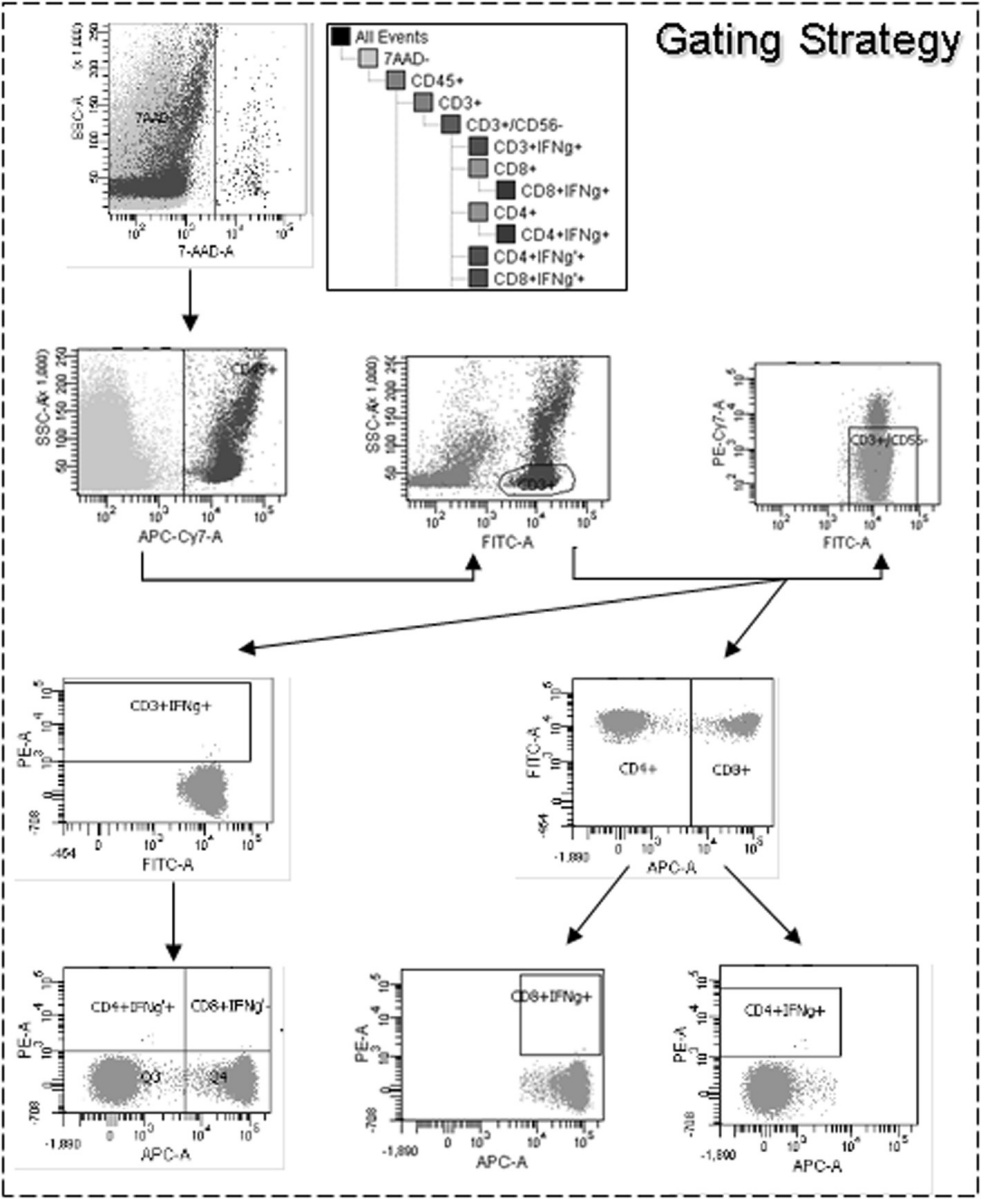
**Gating strategy established for flow cytometric quality and in-process control regarding the CliniMACS CCS validation.** Samples of the collected CliniMACS CCS fraction were analysed by flow cytometry using the Quality control panel QCP-A/A^−^ and the represented gating strategy. All cell fractions (leukapheresis, original fraction (OF), T-cell fraction (TCF), negative fraction (NF), waste fraction (WF), 48 h, 54 h, and 72 h post-leukapheresis (Stabi48, Stabi54, and Stabi72)) were stained with specific antibodies to visualize IFN-γ^+^ T cells. In the first plot, cells were analysed by 7AAD viability staining to determine the live versus dead cells, followed by gating cells based upon CD45 expression to identify CD45^+^ leukocytes in the total viable 7AAD^−^ population. In the next gating step, T cells were selected based on CD3 expression. CD3^+^CD56^+^ NKT cells were gated out using a dump channel. CD4 and CD8 surface expression was then determined from this gated population. IFN-γ^+^ T cells were gated on CD3^+^CD56^−^ T cells and on the CD4^+^ and CD8^+^ subpopulation of CD3^+^CD56^−^ T cells. The axes of the dot plots are biexponential.

Quality control (QC) of the enriched T-cell product and the process-attendant fractions was performed to assess the product characteristics of identity (frequencies of CD3^+^IFN-γ^+/−^ T-cell subsets), viability (total viability, viable leucocytes and lymphocyte subsets), purity (frequencies of contaminating cells), and IFN-γ secretion as marker for potency. Three different marker panels were established (Additional file 2: Table S2). (1) The quality control panel A (QCP-A) was the major quality control panel and was used for the specific identification of viable IFN-γ^+^ T-cell frequencies (Figure 2). The panel consisted of anti-CD45, anti-CD3, anti-CD56, anti-CD8, and anti-IFN-γ mAB. To discriminate unspecific IFN-γ staining a fluorescence minus one control (FMO, QCP-A^−^) was performed. (2) For a detailed purity analysis staining with anti-CD3, anti-CD56, anti-CD14, anti-CD33 and anti-CD19 mAB was established (QCP-B). (3) The BD FACSCantoII flow cytometer is limited to six colours. Therefore anti-CD4 mAb could not be included in the QCP-A, leading to the calculation of CD4^+^ T cells based on the data obtained for CD3^+^ und CD8^+^ T cells. To confirm that this strategy is proper, a third panel (QCP-C) containing anti-CD4 was utilised to proof if by the detection of CD3^+^ and C8^+^ T cells in the QCP-A the correct number of CD4^+^ T cell can calculated. The data proved that staining with anti-CD3 and anti-CD8 is sufficient to reliably separate the CD3^+^CD4^+^ from the CD3^+^CD8^+^ T-cell population. Representative results for the TCF are shown in Additional file 3: Table S3. A mean frequency of 35.1 (range 24–55.9%) CD3^+^CD4^+^ T cells and 25.7% (range 7.23-56.4%) of CD3^+^CD8^+^ T cells was measured with QCP-A, while 34.4% CD3^+^CD4^+^ T cells (range 25–52.3%) and 25.9% (range 7.31-56.7%) of CD3^+^CD8^+^ T cells were quantified by using QCP-C. A notable low standard deviation was calculated between both staining panels for CD3^+^CD4^+^ T cells (range_SD_ 0.25-2.59%) and the CD3^+^CD8^+^ T-cell subpopulation (range_SD_ 0.03-0.22%), respectively. Therefore, all further results shown here were generated by using the results obtained using only the QCP-A. To provide the number of events for a valid quality control without compromising the therapeutic dose, in future processes only QCP-A/A^−^ and QCP-B will be used routinely for in-process and quality control.

All analytic antibodies used for flow cytometry were of *in vitro* diagnostic (IVD) quality. In the process-attendant fractions leukapheresis, OF, and NF at least 50,000 events were acquired in the viable leukocytes gate based on the light scatter properties of leukocytes and their negativity for 7AAD viability staining. Based on low cell numbers in the final TCF and the WF at least 10,000 events (10,000 – 50,000 events) were acquired (Figure 2). Quality control of all collected fractions was performed by using a gating strategy targeted to detect and quantify IFN-γ^+^ T-cell subsets as well as contaminating nonspecific IFN-γ^−^ cells (Figure 2). CD3^+^IFN-γ^+^, CD3^+^IFN-γ^−^, CD8^+^IFN-γ^+^, and CD4^+^IFN-γ^+^ T-cell populations were gated based on the scatter properties of viable T lymphocytes.

### Statistical analysis

Statistical analysis was performed using the Prism software v5.02 (GraphPad, San Diego, California, USA) to analyse the process parameters relevant to quality and the identity, purity, recovery, and viability. The results of the statistical analysis are displayed in the tables and as the mean ± SD in the Figures. Levels of significance were expressed as p-values (*p < 0.05).

## Results

### Verification of CMVpp65-specific T-cell repertoire in preselected T-cell donors from *alloCELL* registry

Three potential CMV-seropositive T-cell donors were recruited from the *alloCELL* registry to validate the manufacturing of clinical-grade CMVpp65-specific T cells (Table 1) according to their CMVpp65 memory T-cell frequencies. Before starting the CliniMACS validation processes, we assessed the data sets of the selected T-cell donor’s CMVpp65-specificity from the *alloCELL* in a detailed analysis (EliSpot assay, CSA, staining of T-cell subsets, A02pp65_M_ staining, Table 1). All three T-cell donors were confirmed and defined to be eligible for T-cell donation by CSA (CMVpp65_pp_, OF_CD3+/IFN-γ+_: mean 3.17%, range 0.21-7.6%, TCF_CD3+/IFN-γ+_: mean 67.8%, range 38.4-89.6%). Leukapheresis products of these healthy T-cell donors were used as starting materials in the validation of the GMP-compliant large-scale enrichment of CMVpp65-specific T cells.

**Table 1:** **Verification of CMV-specific T-cell frequencies in potential T-cell donors selected from the** *alloCELL* **registry**

**Donor**	**HLA-typing**					***alloCELL***		**Verification and detailed analysis of CMV-specific memory T-cell frequencies**						
						**TCR-pMHC interaction**	**EliSpot**	**Staining of T-cell subsets**			**TCR-pMHC interaction**	**CSA**		**EliSpot**
	**A**	**B**	**C**	**DRB1**	**DQB1**	**% A02pp65**_**M**_	**spw**	**% CD3**	**% CD4**	**% CD8**	**% A02pp65**_**M**_	**%OF**	**%TCF**	**spw**
						**[CD19** ^**−**^ **CD3** ^**+**^ **CD8** ^**+**^ **]**	**[IFN-γ** ^**+**^ **]**	**[CD45** ^**+**^ **CD19** ^**−**^ **]**	**[CD3** ^**+**^ **]**	**[CD3** ^**+**^ **]**	**[CD19** ^**−**^ **CD3** ^**+**^ **CD8** ^**+**^ **]**	**[CD3** ^**+**^ **IFN-γ** ^**+**^ **]**	**[CD3** ^**+**^ **IFN-γ** ^**+**^ **]**	**[IFN-γ** ^**+**^ **]**
1	*02:01 *68:01	*08:01 *39:01	*07:01 *12:03	*01:01 *03:01	*02:01 *05:01	2.45	273	78.88	52.47	41.24	1.5	1.7	75.48	236
2	*25:01 *32:01	*08:01 *35:01	*04:01 *07:01	*03:01 *14:01	*02:01 *05:03	n.a.	162	59.65	69.53	26.61	n.a.	0.21	38.39	178.4
3	*02:01 *11:01	*27:02 *55:01	*02:02 *03:03	*15:01 *16:01	*05:02 *06:02	0.31	142	63.79	68.41	26.7	0.34	7.6	89.63	306.5

Three potential T-cell donors we selected according to their CMV-seropositivity and CMVpp65-specific T-cell frequencies from the allogeneic cell registry *alloCELL*. For verification, PBMCs were isolated, T-cell subsets were stained (% T cells [CD3, CD4 and CD8]) and CMVpp65-specific memory T cells were detected directly by pMHC pentamer staining using the A02pp65_M_ (donor 1 and 3, % A02pp65_M_^+^/CD3^+^CD8^+^ T cells) and after a short *ex vivo* stimulation with the CMVpp65_pp_ by IFN-γ-based CSA (% CD3^+^IFN-γ^+^ T cells (OF and TCF) and by IFN-γ EliSpot (spw). Because the donor was HLA-A*02:01 negative = n.a. (not applicable).

### Validation of CMVpp65-specific T-cell enrichment by CliniMACS CCS

**Figure 3:**
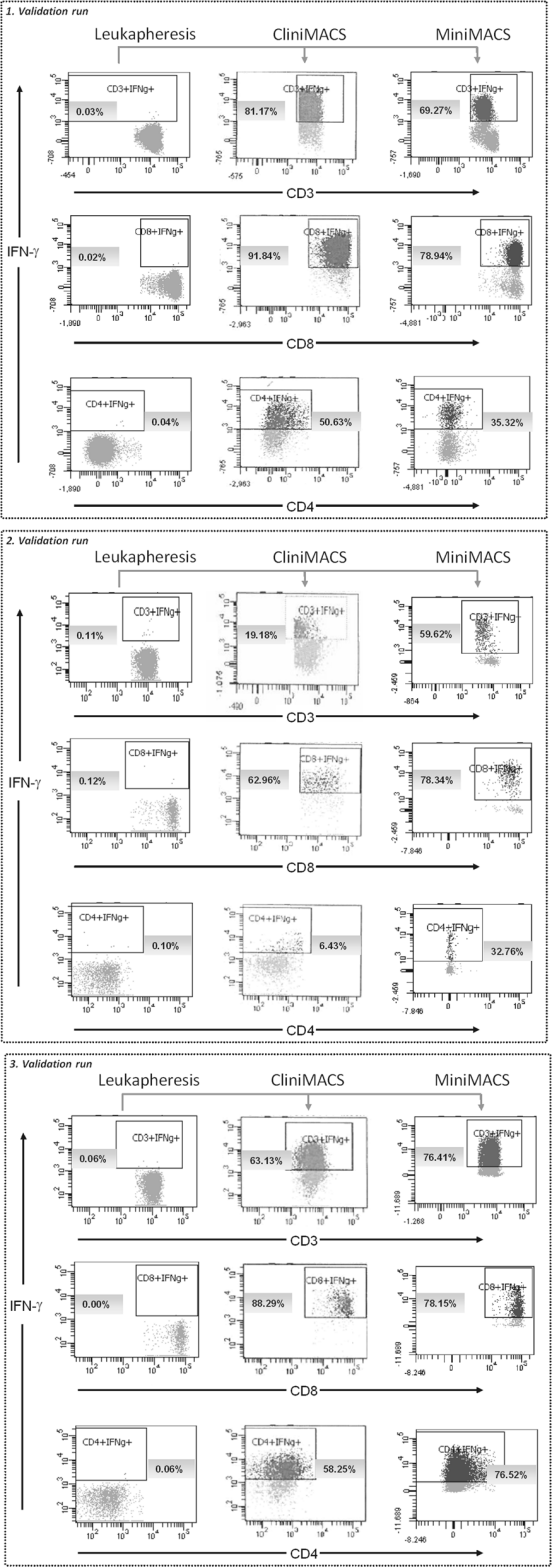
**Flow cytometric quality and in-process control of IFN-γ-based CliniMACS CCS enrichment of CMV-specific T cells.** IFN-γ^+^ CMV-specific T cells were isolated from leukapheresis by large-scale GMP-grade CliniMACS CCS- and small-scale MiniMACS CSA-based process. Flow cytometric analysis was performed with all CliniMACS CCS and MiniMACS CSA fractions (leukapheresis, original fraction (OF), T-cell fraction (TCF), negative fraction (NF), waste fraction (WF), 48 h, 54 h, and 72 h post-leukapheresis (Stabi48, Stabi54, and Stabi72)) by using the quality control panel (QCP) -A, QCP-B and QCP-C-. The results of the representative analysis of the leukapheresis and TCF by using the QCP-A panel are shown (n = 3). As a control the QCP-A^−^ was used as fluorescence minus one (FMO) for IFN-γ. Dot plots show the qualitative analysis of IFN-γ-secreting CMV-specific T cells [%]. CD3^+^IFN-γ^+^ percentages were defined on viable CD3^+^ T cells, and CD8^+^IFN-γ^+^ and CD4^+^IFN-γ^+^ percentages were defined on viable CD4^+^ and viable CD8^+^ T-cells, respectively. IFN-γ^−^secreting T cells are shown in the gate represented on each dot plot.

Each CliniMACS CCS process (n = 3) resulted in the collection of five fractions: leukapheresis, OF, TCF, WF and NF. All leukapheresis averaged 23.9% CD3^+^CD56^−^ T cells (12.8-41.9%; Table 2A-C) with a mean viability of 99.6% (99.3-99.8%). The mean frequency of IFN-γ^+^ T cells 0.07% (0.03-0.11%; Figure 3) indicating no relevant T-cell activation in the native concentrates. Quality control of the OF before enrichment resulted in an IFN-γ^+^ T-cell frequency of 0.76% (range 0.07-1.11%) with a viability of 98.3% (97.9-99.1%, Table 2A-C).

**Table 2:** **Outcome of CMVpp65-specific T-cell separation by CliniMACS CCS**

**A**	***1. Validation run***				
	**Leukapheresis**	**OF**	**TCF**	**WF**	**NF**
volume [ml]	133	100	40	335	276
viability [%]	99.80	99.06	51.13	92.34	99.38
WBCs_(CD45+)_ [x10^6^/ml]	6.77	1.42	0.14	0.02	0.49
WBCs_abs_ [x10^6^]	900.41	142.00	5.51	8.15	136.07
T cells_(CD3+CD56-)_ [% of WBCs]	41.93	47.80	31.68	41.58	44.55
T cells [/μl]	2834.59	680.00	43.50	10.12	219.57
T cells_abs_ [x10^6^]	377.00	68.00	1.74	3.39	60.60
T cells_(CD3+CD4+)_ [% of CD3^+^]	53.00	50.82	26.20	49.74	51.01
T cells_(CD3+CD8+)_ [% of CD3^+^]	47.02	49.20	73.85	50.31	49.01
**IFN-γ** ^**+**^ **T cells [% of CD3** ^**+**^ **]**	**0.03**	**1.02**	**81.17**	**27.13**	**0.63**
IFN-γ^+^ T cells [/μl]	0.85	6.94	35.50	2.74	1.38
**IFN-γ** ^**+**^ **T cells [x10** ^**6**^ **]**	**0.11**	**0.69**	**1.42**	**0.92**	**0.38**
IFN-γ^−^ T cells [% of CD3^+^]	99.97	98.98	18.83	72.87	99.37
IFN-γ^−^ T cells [/μl]	2834.59	673.00	8.20	7.37	218.12
IFN-γ^−^ T cells [x10^6^]	377.00	67.30	0.33	2.47	60.20
IFN-γ^+^ T cells_(CD3+)_ [% of CD4]	0.02	0.29	12.91	6.36	0.17
**IFN-γ** ^**+**^ **T cells**_**(CD3+CD4+)**_ **[% of CD4]**	**0.04**	**0.58**	**50.63**	**13.28**	**0.41**
IFN-γ^+^ T cells_(CD3+CD4+)_ [/μl]	0.60	2.00	5.78	0.67	0.46
**IFN-γ** ^**+**^ **T cells**_**abs (CD3+CD4+)**_ **[x10** ^**6**^ **]**	**0.08**	**0.20**	**0.23**	**0.22**	**0.13**
IFN-γ^+^ T cells_(CD3+)_ [% of CD8]	0.01	0.71	67.96	20.49	0.40
**IFN-γ** ^**+**^ **T cells**_**(CD3+CD8+)**_ **[% of CD8]**	**0.02**	**1.43**	**91.84**	**40.21**	**0.73**
IFN-γ^+^ T cells_(CD3+CD8+)_ [/μl]	0.27	4.79	29.50	2.04	0.79
**IFN-γ** ^**+**^ **T cells**_**abs (CD3+CD8+)**_ **[x10** ^**6**^ **]**	**0.04**	**0.48**	**1.18**	**0.69**	**0.22**
**B**	***2. Validation run***				
	**Leukapheresis**	**OF**	**TCF**	**WF**	**NF**
volume [ml]	114	100	40	337	288
viability [%]	99.31	98.07	62.05	99.27	98.83
WBCs_(CD45+)_ [x10^6^/ml]	18.77	2.36	0.03	0.00	0.66
WBCs_abs_ [x10^6^]	2133.91	236.00	1.06	0.69	190.94
T cells_(CD3+CD56-)_ [% of WBCs]	16.97	36.69	27.92	25.73	41.15
T cells [/μl]	3175.44	865.00	7.43	0.53	272.92
T cells_abs_ [x10^6^]	362.00	86.50	0.30	0.18	78.60
T cells_(CD3+CD4+)_ [% of CD3^+^]	71.57	62.28	78.45	69.19	62.36
T cells_(CD3+CD8+)_ [% of CD3^+^]	28.50	37.75	21.80	30.88	37.71
**IFN-γ** ^**+**^ **T cells [% of CD3** ^**+**^ **]**	**0.11**	**0.07**	**19.18**	**3.80**	**0.01**
IFN-γ^+^ T cells [/μl]	3.50	0.61	1.42	0.02	0.03
**IFN-γ** ^**+**^ **T cells [x10** ^**6**^ **]**	**0.40**	**0.06**	**0.05**	**0.01**	**0.01**
IFN-γ^−^ T cells [% of CD3^+^]	99.89	99.93	80.82	96.20	99.99
IFN-γ^−^ T cells [/μl]	3175.44	864.00	6.00	0.51	272.92
IFN-γ^−^ T cells [x10^6^]	362.00	86.40	0.23	0.17	78.60
IFN-γ^+^ T cells_(CD3+)_ [% of CD4]	0.07	0.02	4.95	1.51	0.01
**IFN-γ** ^**+**^ **T cells**_**(CD3+CD4+)**_ **[% of CD4]**	**0.10**	**0.03**	**6.43**	**1.93**	**0.02**
IFN-γ^+^ T cells_(CD3+CD4+)_ [/μl]	2.27	0.16	0.38	0.01	0.03
**IFN-γ** ^**+**^ **T cells**_**abs (CD3+CD4+)**_ **[x10** ^**6**^ **]**	**0.26**	**0.02**	**0.02**	**0.00**	**0.01**
IFN-γ^+^ T cells_(CD3+)_ [% of CD8]	0.04	0.04	13.84	2.18	0.00
**IFN-γ** ^**+**^ **T cells**_**(CD3+CD8+)**_ **[% of CD8]**	**0.12**	**0.10**	**62.96**	**6.52**	**0.00**
IFN-γ^+^ T cells_(CD3+CD8+)_ [/μl]	1.09	0.32	1.02	0.01	0.00
**IFN-γ** ^**+**^ **T cells**_**abs (CD3+CD8+)**_ **[x10** ^**6**^ **]**	**0.12**	**0.03**	**0.04**	**0.00**	**0.00**
**C**	***3. Validation run***				
	**Leukapheresis**	**OF**	**TCF**	**WF**	**NF**
volume [ml]	109	100	43	330	263
viability [%]	99.56	97.86	58.92	90.43	98.21
WBCs_(CD45+)_ [x10^6^/ml]	15.73	1.62	0.12	0.01	0.53
WBCs_abs_ [x10^6^]	1714.31	162.00	5.01	4.82	138.49
T cells_(CD3+CD56-)_ [% of WBCs]	12.76	30.94	36.52	31.07	28.56
T cells [/μl]	2009.17	500.00	42.56	4.55	150.19
T cells_abs_ [x10^6^]	219.00	50.00	1.83	1.50	39.50
T cells_(CD3+CD4+)_ [% of CD3^+^]	75.96	72.60	87.29	80.31	70.89
T cells_(CD3+CD8+)_ [% of CD3^+^]	24.08	27.43	12.81	19.71	29.13
**IFN-γ** ^**+**^ **T cells [% of CD3** ^**+**^ **]**	**0.06**	**1.11**	**63.13**	**36.29**	**0.47**
IFN-γ^+^ T cells [/μl]	1.20	5.55	26.74	1.65	0.71
**IFN-γ** ^**+**^ **T cells [x10** ^**6**^ **]**	**0.13**	**0.56**	**1.15**	**0.54**	**0.19**
IFN-γ^−^ T cells [% of CD3^+^]	99.94	98.89	36.87	63.71	99.53
IFN-γ^−^ T cells [/μl]	2009.17	494.00	15.67	2.89	149.81
IFN-γ^−^ T cells [x10^6^]	219.00	49.40	0.67	0.95	39.40
IFN-γ^+^ T cells_(CD3+)_ [% of CD4]	0.06	0.87	53.17	30.37	0.47
**IFN-γ** ^**+**^ **T cells**_**(CD3+CD4)**_ **[% of CD4]**	**0.06**	**1.10**	**58.25**	**34.13**	**0.49**
IFN-γ^+^ T cells_(CD3+CD4+)_ [/μl]	0.91	3.99	21.63	1.24	0.52
**IFN-γ** ^**+**^ **T cells**_**abs (CD3+CD4+)**_ **[x10** ^**6**^ **]**	**0.10**	**0.40**	**0.93**	**0.41**	**0.14**
IFN-γ^+^ T cells_(CD3+)_ [% of CD8]	0.02	0.30	11.48	7.75	0.10
**IFN-γ** ^**+**^ **T cells**_**(CD3+CD8+)**_ **[% of CD8]**	**0.00**	**0.99**	**88.29**	**37.27**	**0.30**
IFN-γ^+^ T cells_(CD3+CD8+)_ [/μl]	0.00	1.36	4.81	0.33	0.13
**IFN-γ** ^**+**^ **T cells**_**abs (CD3+CD8+)**_ [*10 ^**6**^ **]**	**0.00**	**0.14**	**0.21**	**0.11**	**0.03**

The purification of clinical-grade CMVpp65-specific CD4^+^ and CD8^+^ T cells from three healthy CMV-seropositive donors was performed aseptically under GMP conditions using the IFN-γ-based CliniMACS CCS system and the GMP-compliant CMVpp65_pp_ for short term *ex vivo* stimulation. Detailed information for all three validation processes (A-B) including viability [%], viable cell number [x10^6^/ml; x10^6^; /μl] and specific cell frequencies [%] for all CliniMACS CCS fractions were determined. The results for the representative analysis of the cells from the leukapheresis, original fraction (OF), T-cell fraction (TCF), negative fraction (NF), and waste fraction (WF) are shown. Bold data reflected the results obtained for the CMV-specific IFN-γ-positive T cells.

**Figure 4:**
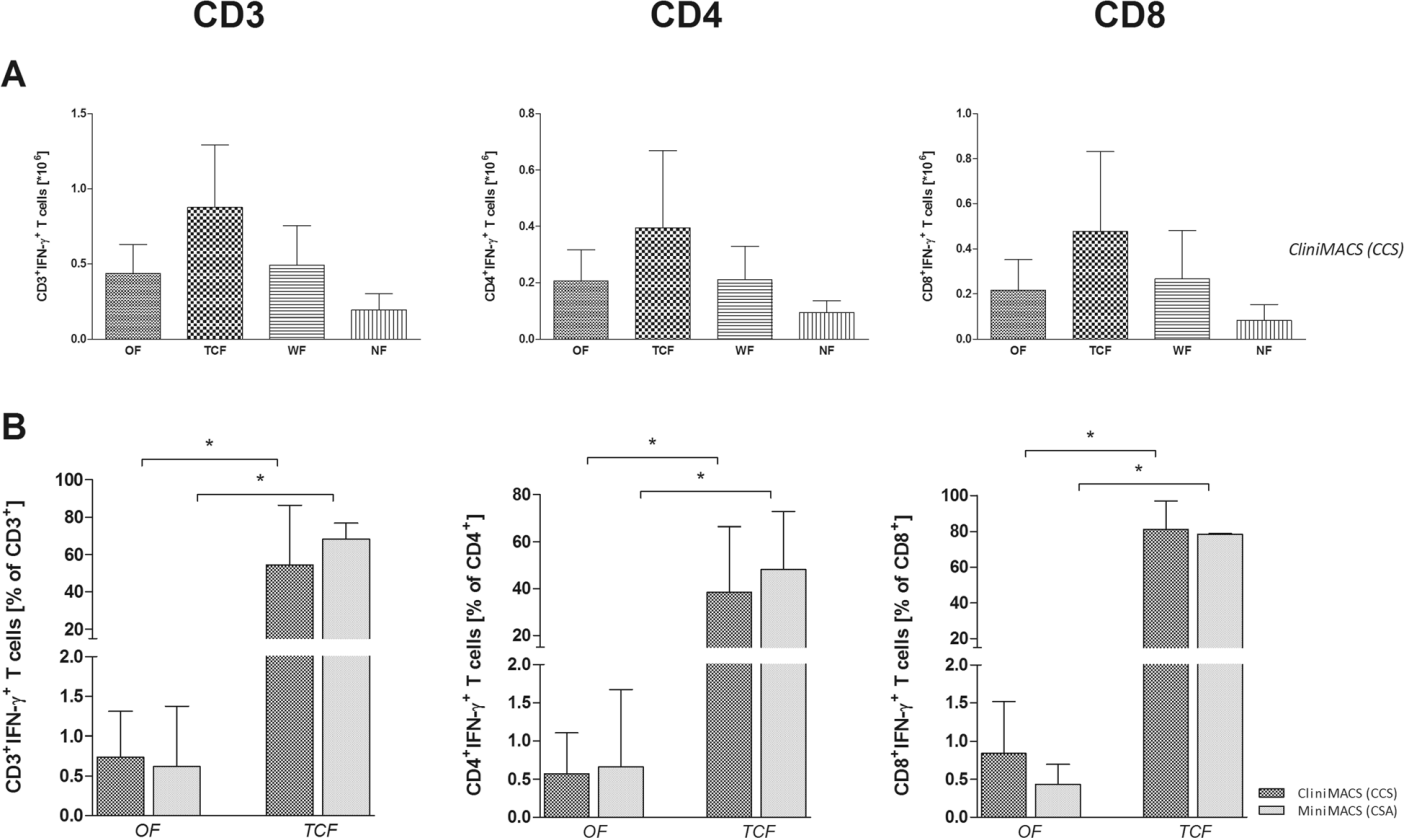
**Efficiency and outcomes of CliniMACS CCS validation for CMVpp65-specific T-cell enrichment.** The percentage of IFN-γ secreting CMVpp65-specific T cells was detected after four hours of *ex vivo* stimulation with CMVpp65_pp_ using the QCP-A/A^-^ panel. The IFN-γ-based CliniMACS CCS and MiniMACS CSA systems were used for the isolation of IFN-γ-secreting CMVpp65-specific T cells. **(A)** The numbers of IFN-γ^+^ cells [x10^6^] within the CD3, CD4 and CD8 T-cell populations were analysed in all collected CliniMACS CCS fractions (leukapheresis, original fraction (OF), T-cell fraction (TCF), negative fraction (NF), waste fraction (WF), 48 h, 54 h, and 72 h post-leukapheresis (Stabi48, Stabi54, Stabi72)) to determine the efficiency of the CliniMACS process. Data sets of the representative analysis of the leukapheresis, OF, TCF, NF, WF fractions are shown **(B)** Outcome of IFN-γ-based CliniMACS CCS and MiniMACS CSA processes regarding the percentage [%] of IFN-γ^+^ cells among the CD3, CD4 and CD8 T-cell populations in samples collected at different steps of the validation process (leukapheresis, OF, TCF, NF, WF, Stabi48, Stabi54, Stabi72). Data sets of the representative analysis of the leukapheresis and TCF are shown. The results of independent experiments (n = 3) are expressed as the mean frequency of IFN-γ^+^ T cells ± SD. Asterisks indicate statistically significant differences between samples before and after T-cell enrichment (*p < 0.05).

The TCF of the three validation runs contained 19.2-81.2% CD3^+^IFN-γ^+^ T cells (0.05-1.42 × 10^6^, mean 0.87 × 10^6^) with a viability of 57.4% (range 51.1-62.1%; Table 2, Figures 3 and 4A) in a total volume of 40–43 ml. A frequency of 18.8-80.8% contaminating, potentially alloreactive CD3^+^IFN-γ^−^ T cells (0.23-0.67 × 10^6^, mean 0.41 × 10^6^) was calculated. In relation to the number of CD3^+^IFN-γ^+^ T cells determined in the OF, a 213-fold decrease (range 73-369-fold) was observed in the TCF.

For the analysis of the enrichment efficiency by CliniMACS CCS, the recovery of total CD3^+^IFN-γ^+^ T cells, CD4^+^IFN-γ^+^ T cells and CD8^+^IFN-γ^+^ T cells (Figure 4B, Table 3) was calculated based on the percentage of IFN-γ^+^ T cells in the CMVpp65_pp_-stimulated OF and the enriched TCF. The recovery of total CD3^+^IFN-γ^+^ T cells post-enrichment averaged 67.9 ± 22.7% (CD4^+^ IFN-γ^+^ T-cell recovery: 68.8 ± 57.2%, CD8^+^IFN-γ^+^ T-cell recovery: 57.2 ± 23.4%). Furthermore the CMVpp65-specific TCF contained a mean of 54.5 ± 31.9% IFN-γ^+^ T cells with a percentage of 38.4 ± 28% CD4^+^IFN-γ^+^ and 81 ± 15.8% CD8^+^IFN-γ^+^ T cells. It was shown that 1 × 10^4^ CD3^+^ T cells per kg body weight were efficient for adoptive transfer [8]. According to this, CliniMACS CCS enrichment resulted in a sufficient number of total CD3^+^CD56^−^ T cells as well as total CD3^+^IFN-γ^+^ T cells for adoptive transfer in recipients up to 183 kg of body weight (validation run 3). Moreover, the percentage of CD8^+^IFN-γ^+^ T cells was higher than that of CD4^+^IFN-γ^+^ T cells (Figure 3A-C; Table 2A-C) in all three performed CliniMACS CCS validation runs.

**Table 3:** **Overall outcome of the CliniMACS CCS validation process for the manufacture of CMVpp65-specific T cells**

***n = 3***	***%CD3*** ^***+***^ ***IFN-γ*** ^***+***^ ***recovery***	***%CD4*** ^***+***^ ***IFN-γ*** ^***+***^ ***recovery***	***%CD8*** ^***+***^ ***IFN-γ*** ^***+***^ ***recovery***	***%CD3*** ^***+***^ ***IFN-γ*** ^***+***^ ***purity***	***%CD4*** ^***+***^ ***IFN-γ*** ^***+***^ ***purity***	***%CD8*** ^***+***^ ***IFN-γ*** ^***+***^ ***purity***	***% total viability***
*Mean*	67.86	68.81	57.20	54.49	38.44	81.03	57.37
*SD*	22.66	57.20	23.42	31.88	27.98	15.75	1.61
*Median*	77.69	69.95	70.37	63.13	50.63	88.29	58.92
*Min*	41.94	11.04	30.16	19.18	6.43	62.96	51.13
*Max*	83.94	125.43	71.08	81.17	58.25	91.84	62.05

The recovery of IFN-γ^+^ T cells [%] in the products after large-scale CliniMACS CCS enrichment was calculated based on the CMVpp65_pp_-stimulated original fraction (OF) and the final collected fractions (T-cell fraction (TCF), waste fraction (WF), negative fraction (NF), TCF after 48 h, 54 h, and 72 h post-leukapheresis (Stabi48, Stabi54, Stabi72)) as recovered from the CliniMACS tubing set. The result for the representative analysis of the recovery from the TCF is shown. The purity of IFN-γ^+^ T cells post-CliniMACS CCS enrichment was calculated as the percentage of CD3^+^CD56^−^ lymphocytes [%]. Total viability was assessed by 7AAD viability staining.

As expected, a significantly lower number of IFN-γ^+^ T cells (frequency: 0.01-0.63%; total cell count: 0.01-0.38 × 10^6^; Figure 4A, Table 2) was found in the NF compared to the respective TCF of all three runs. The viability in the NF approximated 100% (range 98.2-99.4%). During the process IFN-γ^+^ T cells were lost in the WF in a much higher frequency than expected (mean viability 94%; frequency IFN-γ^+^ T cells: 3.8-36.3%, 0.01-0.92 × 10^6^; Figure 4A, Table 2). Leukapheresis products and TCFs of the three CliniMACS CCS validation runs did not show contamination assessed by aerobic and anaerobic cultures. Overall, the specific risk-based acceptance criteria (Additional file 1: Table S1) were fulfilled in all validation runs.

### Stability evaluation of CliniMACS CCS-enriched T-cell products

**Figure 5:**
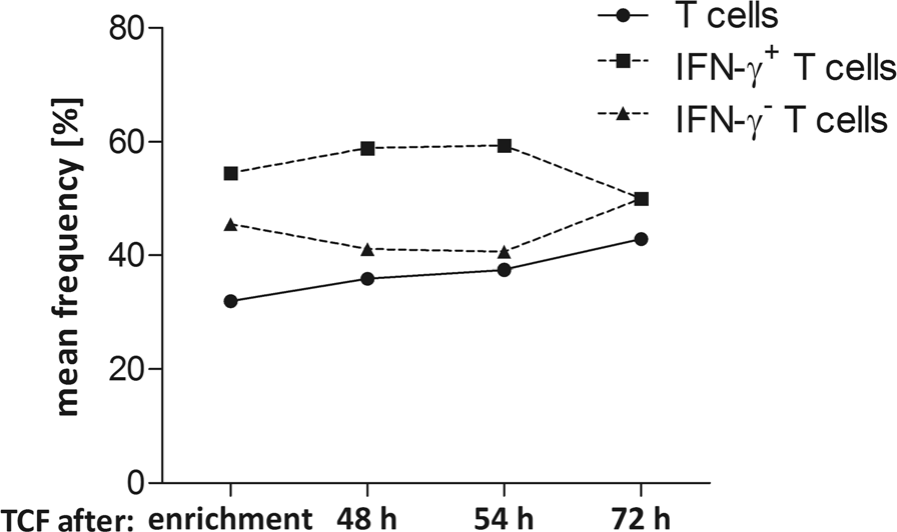
**Analysis of product stability.** Stability of the TCF was analysed after 48 h, 54 h and 72 h of the start of leukapheresis with respect to product viability [%], frequency of CD3^+^CD56^−^ T cells [%] in CD45^+^ leukocytes and IFN-γ^+/−^ T cells [%] in CD3^+^CD56^−^ lymphocytes. The results of independent experiments are expressed as the mean frequency [%] of viability, T cells and IFN-γ^+/−^ T cells with regard to the different time points of storage.

To determine the shelf life of the CMVpp65-specific TCF, aliquots were stored in CliniMACS PBS/EDTA buffer supplemented with 0.5% HSA over a total of 72 h after leukapheresis at 2-6°C in the target fraction bag of the CliniMACS tubing set as the primary container and analysed kinetically (Table 4). The average recovery of viability of stored TCFs was >100% for each defined time point. Overall, a total of 4.57 × 10^6^ viable leukocytes (viable WBCs, range 3.6-6.2 × 10^6^) with an average recovery of 75% and a total of 1.19 × 10^6^ viable CD3^+^CD56^−^ T cells (viable T cells, range 0.25-2.09 × 10^6^) with an average recovery of 85% were found after 72 h post-leukapheresis. The frequencies of CD3^+^CD56^−^ T cells as well as CD3^+^CD56^−^IFN-γ^+/−^ T cells were stable (Figure 5). Stability analysis resulted in a recovery rate of 118% for viable CD3^+^CD56^−^ (79.48 to >100%), 120% for viable CD3^+^CD56^−^IFN-γ^+^ T cells (84.25 to >100%), and viable 109% for CD3^+^CD56^−^IFN-γ^−^ T cells (77.12 to >100%). TCFs might contain cells which are still intact or on the verge of apoptosis. During storage those cells may be lost because of cell lysis resulting in recovery rates >100%.

**Table 4:** **Analysis of product stability**

**Parameter**	**Validation run**	**TCF after enrichment**	**48 h**		**54 h**		**72 h**	
			**value**	**recovery**	**value**	**recovery**	**value**	**recovery**
WBC	1. run	1.43x10^7^	9.60x10^6^	67.00%	7.80x10^6^	54.50%	7.60x10^6^	53.10%
	2. run	5.00x10^6^	4.40x10^6^	88.00%	4.20x10^6^	84.00%	4.00x10^6^	80.00%
	3. run	1.25x10^7^	8.60x10^6^	69.00%	7.30x10^6^	58.60%	6.50x10^6^	51.70%
viable WBC	1. run	1.01x10^7^	8.00x10^6^	79.50%	7.20x10^6^	71.60%	6.20x10^6^	61.70%
	2. run	3.40x10^6^	3.20x10^6^	94.10%	3.80x10^6^	>100%	3.60x10^6^	>100%
	3. run	6.00x10^6^	5.20x10^6^	85.70%	4.30x10^6^	71.40%	3.90x10^6^	64.30%
viable T cells	1. run	1.74x10^6^	1.42x10^6^	81.61	1.32x10^6^	75.86%	1.22x10^6^	70.11%
	2. run	2.97x10^6^	2.17x10^5^	73.06%	1.97x10^5^	66.33%	2.49x10^5^	83.84%
	3. run	1.83x10^6^	2.12x10^6^	>100%	1.85x10^6^	>100%	2.09x10^6^	>100%

Stability of the cells from the T-cell fraction (TCF) was analysed after 48 h, 54 h and 72 h post-leukapheresis with respect to total numbers of WBCs [x10^6^], viable WBCs [x10^6^] viable T cells (CD3^+^CD56^−^ T cells) [x10^6^], and recovery [%]. Detection of total cell numbers and viability was performed by light microscopy using trypan blue dye.

### Enrichment of CMVpp65-specific T cells by non-GMP MiniMACS CSA as a control process

The analogous parallel processing of CMVpp65-specific T cells by non-GMP small-scale MiniMACS CSA resulted in CD3^+^IFN-γ^+^ T-cell yields similar to those observed in the CliniMACS CCS processes (Figures 3 and 4B, Tables 2 and 5). The first (69.3%, viability 15.6%; Figure 3A, Table 5A) and the third (76.4%, viability 45.8%; Figure 3C, Table 5C) MiniMACS CSA process resulted in ratios of IFN-γ^+^ T cells comparable to those established by clinical-scale CliniMACS CCS. In the second CliniMACS CCS validation run, a significantly lower percentage of IFN-γ^+^ T cells was obtained in comparison to the small-scale MiniMACS CSA (19.2% vs. 59.6%; viability 62.1% vs. 85.2%; Figure 3B, Tables 2B and 5B). In addition to CMVpp65_pp_-restimulated T cells, SEB-stimulated (PC, positive control) and unstimulated (NC, negative control) T cells were used as controls in the small-scale MiniMACS CSA (Table 5). Negative controls resulted in significantly lower numbers of isolated IFN-γ^+^ T cells (range 1.56-3.85%; viability 12.2-30%) compared to the positive controls (range 60.8-82.3%, viability 33.2-89%).

**Table 5:** **Outcome of CMVpp65-specific T-cell separation by MiniMACS CSA**

**A**	***1. Validation run***					
	**Positive control**		**CMVpp65**_**pp**_		**Negative control**	
	**OF**	**TCF**	**OF**	**TCF**	**OF**	**TCF**
volume [ml]	0.6	1.0	0.6	1.0	0.6	1.0
viability [%]	98.81	33.19	98.70	15.63	98.67	12.21
WBCs_(CD45+)_ [x10^6^/ml]	42.70	0.07	47.50	0.01	39.50	0.00
WBCs_abs_ [x10^6^]	25.62	0.07	28.50	0.01	23.70	0.00
T cells_(CD3+CD56-)_ [% of WBCs]	27.26	35.72	27.56	43.33	31.66	15.62
T cells [/μl]	11650.00	24.90	13100.00	6.23	12500.00	0.70
T cells_abs_ [x10^6^]	6.99	0.02	7.86	0.01	7.50	0.00
T cells_(CD3+CD4+)_ [% of CD3^+^]	50.34	33.51	50.25	25.85	49.05	63.35
T cells_(CD3+CD8+)_ [% of CD3^+^]	49.67	66.51	49.77	74.15	50.97	36.71
**IFN-γ** ^**+**^ **T cells [% of CD3** ^**+**^ **]**	**0.69**	**82.33**	**0.23**	**69.27**	**0.01**	**2.89**
IFN-γ^+^ T cells [/μl]	80.33	20.50	30.17	4.32	1.25	0.02
**IFN-γ** ^**+**^ **T cells [x10** ^**4**^ **]**	**4.74**	**1.21**	**1.79**	**0.15**	**0.07**	**0.00**
IFN-γ^−^ T cells [% of CD3^+^]	99.31	17.67	99.77	30.73	99.99	97.11
IFN-γ^−^ T cells [/μl]	11566.67	4.40	13066.67	1.92	12500.00	0.68
IFN-γ^−^ T cells [x10^4^]	682	026	778	0.07	724	0.02
IFN-γ^+^ T cells_(CD3+)_ [% of CD4]	0.18	23.44	0.04	9.06	0.01	1.72
**IFN-γ** ^**+**^ **T cells**_**(CD3+CD4+)**_ **[% of CD4]**	**0.37**	**70.45**	**0.09**	**35.32**	**0.01**	**2.82**
IFN-γ^+^ T cells_(CD3+CD4+)_ [/μl]	21.67	5.88	5.93	0.57	0.61	0.01
**IFN-γ** ^**+**^ **T cells**_**abs (CD3+CD4+)**_ **[x10** ^**4**^ **]**	**1.28**	**0.35**	**0.35**	**0.02**	**0.04**	**0.00**
IFN-γ^+^ T cells_(CD3+)_ [% of CD8]	0.49	58.79	0.18	59.14	0.00	0.52
**IFN-γ** ^**+**^ **T cells**_**(CD3+CD8+)**_ **[% of CD8]**	**0.95**	**87.52**	**0.37**	**78.94**	**0.00**	**1.42**
IFN-γ^+^ T cells_(CD3+CD8+)_ [/μl]	55.00	14.50	24.17	3.65	0.00	0.00
**IFN-γ** ^**+**^ **T cells**_**abs (CD3+CD8+)**_ **[x10** ^**4**^ **]**	**3.24**	**0.86**	**1.44**	**0.13**	**0.00**	**0.01**
**B**	***2. Validation run***					
	**Positive control**		**CMVpp65**_**pp**_		**Negative control**	
	**OF**	**TCF**	**OF**	**TCF**	**OF**	**TCF**
volume [ml]	0.6	1.0	0.6	1.0	0.6	1.0
viability [%]	98.72	89.04	98.92	85.16	98.87	29.95
WBCs_(CD45+)_ [x10^6^/ml]	2.38	0.18	2.74	0.00	2.78	0.00
WBCs_abs_ [x10^6^]	1.43	0.18	1.64	0.00	1.67	0.00
T cells_(CD3+CD56-)_ [% of WBCs]	95.94	88.43	89.37	68.87	88.83	55.91
T cells [/μl]	2283.33	162.00	2450.00	1.42	2466.67	11.10
T cells_abs_ [x10^6^]	1.37	0.16	1.47	0.00	1.48	0.00
T cells_(CD3+CD4+)_ [% of CD3^+^]	71.87	75.54	71.00	43.38	69.45	71.15
T cells_(CD3+CD8+)_ [% of CD3^+^]	28.36	24.51	29.06	56.73	30.59	28.85
**IFN-γ** ^**+**^ **T cells [% of CD3** ^**+**^ **]**	**5.74**	**60.77**	**0.13**	**59.62**	**0.02**	**3.85**
IFN-γ^+^ T cells [/μl]	131.00	98.30	3.18	0.85	0.49	0.43
**IFN-γ** ^**+**^ **T cells [x10** ^**4**^ **]**	**7.86**	**9.83**	**0.19**	**0.08**	**0.03**	**0.00**
IFN-γ^−^ T cells [% of CD3^+^]	94.26	39.23	99.87	40.38	99.98	96.15
IFN-γ^−^ T cells [/μl]	2150.00	63.50	2450.00	0.57	2466.67	10.70
IFN-γ^−^ T cells [x10^4^]	129	6.35	147	0.06	148	0.11
IFN-γ^+^ T cells_(CD3+)_ [% of CD4]	3.83	43.77	0.07	14.53	0.02	1.92
**IFN-γ** ^**+**^ **T cells**_**(CD3+CD4+)**_ **[% of CD4]**	**4.99**	**56.07**	**0.06**	**32.76**	**0.03**	**2.70**
IFN-γ^+^ T cells_(CD3+CD4+)_ [/μl]	81.83	68.50	1.04	0.20	0.52	0.21
**IFN-γ** ^**+**^ **T cells**_**abs (CD3+CD4+)**_ **[x10** ^**4**^ **]**	**4.91**	**6.85**	**0.06**	**0.02**	**0.03**	**0.00**
IFN-γ^+^ T cells_(CD3+)_ [% of CD8]	1.80	16.45	0.06	45.09	0.00	1.92
**IFN-γ** ^**+**^ **T cells**_**(CD3+CD8+)**_ **[% of CD8]**	**5.75**	**64.53**	**0.21**	**78.34**	**0.00**	**6.67**
IFN-γ^+^ T cells_(CD3+CD8+)_ [/μl]	37.17	25.60	1.50	0.63	0.00	0.21
**IFN-γ** ^**+**^ **T cells**_**abs (CD3+CD8+)**_ **[x10** ^**4**^ **]**	**2.23**	**2.56**	**0.09**	**0.06**	**0.00**	**0.00**
**C**	***3. Validation run***					
	**Positive control**		**CMVpp65**_**pp**_		**Negative control**	
	**OF**	**TCF**	**OF**	**OF**	**TCF**	**OF**
volume [ml]	0.6	1.0	0.6	1.0	0.6	1.0
viability [%]	98.57	69.24	98.87	45.75	98.63	26.57
WBCs_(CD45+)_ [x10^6^/ml]	4.23	0.02	3.33	0.01	3.66	0.00
WBCs_abs_ [x10^6^]	2.54	0.02	2.00	0.01	2.20	0.00
T cells_(CD3+CD56-)_ [% of WBCs]	98.13	94.39	99.99	95.45	95.87	73.00
T cells [/μl]	4150.00	21.20	3333.33	11.90	3516.67	0.00
T cells_abs_ [x10^6^]	2.49	0.02	2.00	0.01	2.11	0.00
T cells_(CD3+CD4+)_ [% of CD3^+^]	72.20	66.59	72.07	88.51	68.63	84.38
T cells_(CD3+CD8+)_ [% of CD3^+^]	27.80	33.41	27.93	11.49	31.37	15.62
**IFN-γ** ^**+**^ **T cells [% of CD3** ^**+**^ **]**	**2.73**	**70.70**	**1.49**	**76.41**	**0.00**	**1.56**
IFN-γ^+^ T cells [/μl]	113.33	15.00	49.50	9.06	0.00	0.00
**IFN-γ** ^**+**^ **T cells [x10** ^**4**^ **]**	**6.80**	**1.50**	**2.97**	**0.91**	**0.00**	**0.00**
IFN-γ^−^ T cells [% of CD3^+^]	97.27	29.30	98.51	23.59	100.00	98.44
IFN-γ^−^ T cells [/μl]	4033.33	6.22	3283.33	2.80	3516.67	0.00
IFN-γ^−^ T cells [x10^4^]	242	0.62	197	0.28	211	0.00
IFN-γ^+^ T cells_(CD3+)_ [% of CD4]	1.55	45.27	1.35	68.14	0.00	0.76
**IFN-γ** ^**+**^ **T cells**_**(CD3+CD4+)**_ **[% of CD4]**	**2.17**	**67.26**	**1.83**	**76.52**	**0.00**	**1.04**
IFN-γ^+^ T cells_(CD3+CD4+)_ [/μl]	65.00	9.50	43.83	8.03	0.00	0.00
**IFN-γ** ^**+**^ **T cells**_**abs (CD3+CD4+)**_ **[x10** ^**4**^ **]**	**3.90**	**0.95**	**2.63**	**0.80**	**0.00**	**0.00**
IFN-γ^+^ T cells_(CD3+)_ [% of CD8]	1.19	26.20	0.18	8.80	0.00	0.52
**IFN-γ** ^**+**^ **T cells**_**(CD3+CD8+)**_ **[% of CD8]**	**4.33**	**81.11**	**0.72**	**78.15**	**0.00**	**3.33**
IFN-γ^+^ T cells_(CD3+CD8+)_ [/μl]	50.00	5.75	6.68	1.06	0.00	0.00
**IFN-γ** ^**+**^ **T cells**_**abs (CD3+CD8+)**_ **[x10** ^**4**^ **]**	**3.00**	**0.58**	**0.40**	**0.11**	**0.00**	**0.00**

The small-scale MiniMACS CSA was performed as a control for the large-scale CliniMACS CCS process from the same leukapheresis. Detailed information for all three MiniMACS CSA processes (A-B) including viability [%], viable cell number [x10^6^/ml; x10^6^; /μl] and specific cell frequencies [%] for all CSA fractions was determined. The results for the representative analysis of the cells from the original fraction (OF) and T-cell fraction (TCF) are shown. As a positive control, cells were stimulated with SEB, while cells cultured in medium alone served as the negative control; CMVpp65pp = CMVpp65 peptide pool. Bold data reflected the results obtained for the antigen-specific IFN-γ-positive T cells.

### Composition of leukocyte subsets of CliniMACS CCS fractions

**Figure 6:**
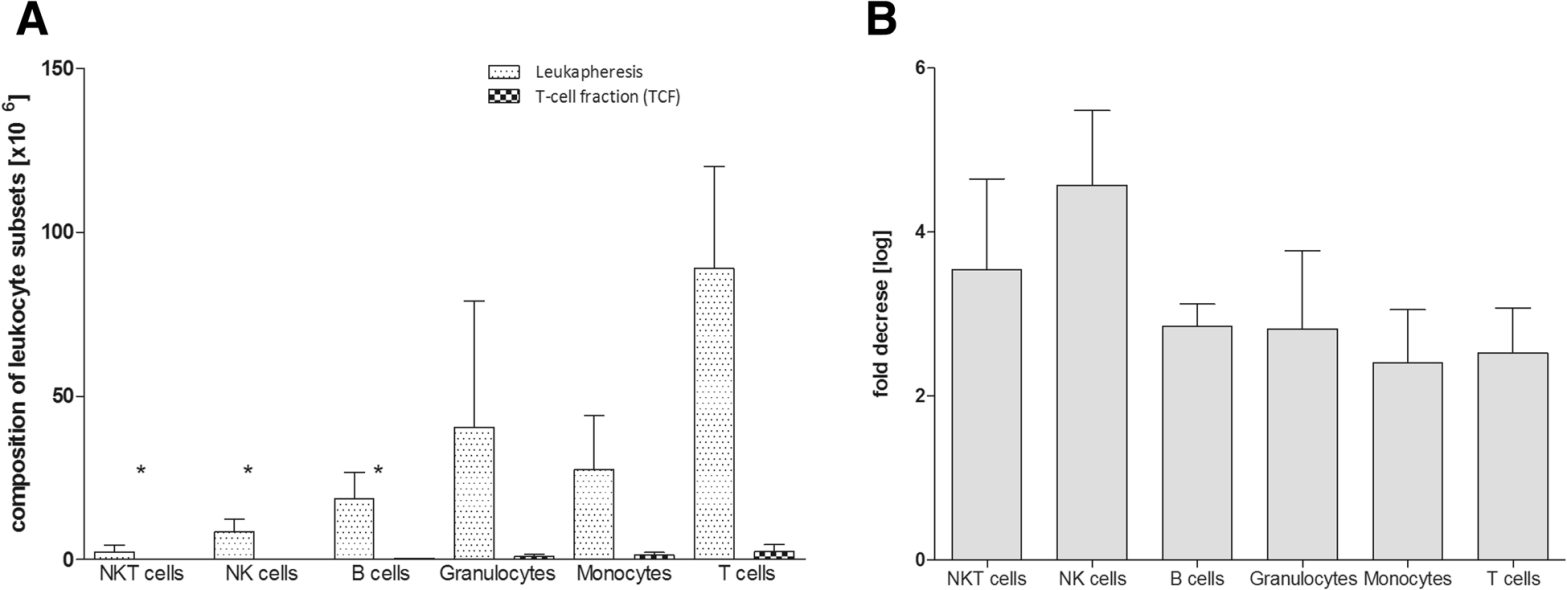
**Post-processing assessment of leukocyte subsets in the TCF.** Fractions collected during the CliniMACS CCS process (leukapheresis, original fraction (OF), T-cell fraction (TCF), negative fraction (NF), waste fraction (WF), 48 h, 54 h, and 72 h post-leukapheresis (Stabi48, Stabi54, Stabi72)) were assessed for leukocyte subsets including: CD3^+^ T cells, CD3^+^CD56^+^ NKT cells, CD3^−^CD56^+^ NK cells, CD19^+^ B cells, CD33^+^ granulocytes and CD14^+^ monocytes. **(A)** The compositions of the leukocyte subsets in the Leukapheresis products and the TCFs and **(B)** the log depletion in cell numbers of leukocyte subsets after CliniMACS CCS enrichment are shown. The results of independent experiments are expressed as the number [x10^6^] of IFN-γ^+^ T cells ± SD and the fold decrease [log] of cell numbers in leukocyte subsets. Asterisks indicate statistically significant differences between T cells and other leukocyte subsets (*p < 0.05).

The composition of all fractions collected during the CliniMACS CCS processes was evaluated in depth with respect to the content of total CD3^+^, CD14^+^, CD19^+^, CD33^+^, and CD56^+^ leukocytes using the QCP-B (Figure 6). The most infrequent contaminants in the TCF were CD3^+^CD56^+^ NKT cells (mean 0.22 × 10^5^) and CD3^−^CD56^+^ NK cells (mean 0.11 × 10^5^). Furthermore, CD14^+^ monocytes (mean 1.46 × 10^6^_)_, CD33^+^ granulocytes (mean 0.9 × 10^6^), and CD19^+^ B cells (mean 0.29 × 10^6^) represented the most common non-target cells (Figure 6A). Nevertheless, the total number of these subsets was decreased compared to the initial leukapheresis. Highest log-depletion was observed for NK cells (4.57-fold) and NKT cells (3.54-fold), respectively. B-cell number decreased 2.85-fold, followed by granulocytes (2.82-fold), CD3^+^CD56^−^ T cells (2.52-fold), and monocytes (2.41-fold) (Figure 6B).

## Discussion

A three-step protocol (Figure 1) for the rapid generation of clinical-grade antiviral T cells was validated to facilitate the manufacture of specific T-cells, thereby allowing a pre-emptive or prophylactic adoptive T-cell transfer.

### First step (Selection of potential T-cell donors from alloCELL)

The efficacy and safety of partially HLA-mismatched third-party antiviral T-cell transfer has been demonstrated in a number of studies, making the recruitment of these donors a useful option for the effective treatment of life-threatening infections or reactivation of common viruses such as CMV, EBV, ADV, or HHV6 [2,3,5-8,16,26]. The *alloCELL* registry established at the Hannover Medical School represents a flexible platform which facilitates the rapid identification and recruitment of adequate donors according to HLA type, virus serology and virus-specific T-cell response [19]. It currently provides data sets of memory T-cell frequencies of more than 450 possible T-cell donors achieved by IFN-γ-based immunoassays EliSpot, ICS and CSA as well as by specific TCR staining using pMHC multimers [19,25].

### Second step (Verification of the donor’s specific T-cell frequencies and prediction of the donor’s T-cell enrichment efficiency by MiniMACS CSA)

In previous laboratory-scale MiniMACS CSA experiments using CMVpp65_pp_, we demonstrated that donors falling below a critical starting frequency of target cells most likely result in a low purity of the enriched target cell fraction. Therefore, donor eligibility (stem cell donor, family donor as well as third party donor) was subjected to lower limits of: (a) the peripheral frequency of virus-specific IFN-γ^+^ T cells (≥0.03% of total CD3^+^ T cells) and of (b) the restimulation efficiency (twice as much as the unstimulated control). We successfully yielded highly pure CMVpp65-specific T cells from recruited donors (Table 1), confirming the validity of data provided by the *alloCELL* registry and the criteria for T-cell donor eligibility.

### Third step (Manufacturing of clinical-grade antiviral T cells by CliniMACS CCS)

The enrichment results determined in our study can strongly be compared with recent studies demonstrating the successful manufacture of highly pure antigen-specific T cells by IFN-γ based CliniMACS CCS [3,6,7,27]. We yielded IFN-γ^+^ T cells with a mean purity of 54.5 ± 31.9%, which is comparable to data published for the enrichment of CMV- (43.9-65% purity), EBV- (57% purity) and ADV-specific (52–63.4% purity) T cells using the CliniMACS CCS [1,3,4,6,8]. The quality of the final TCFs was successfully demonstrated by (1) IFN-γ secretion as potency marker, (2) extensive flow cytometric quality control, and (3) stability testing of the TCFs.

### Manufacture of clinical-grade antiviral T cells by large-scale CliniMACS CCS

As an eligibility requirement for T-cell donor selection the lower limit of the peripheral frequency of CMV-specific IFN-γ^+^ T cells was defined with ≥0.03% of total CD3^+^ T cells. For a variety of viruses including EBV, ADV, and HHV6 the circulating precursor frequency of reactive cells will likely be substantially lower than for CMV. Therefore, the threshold of IFN-γ^+^ T cells has to be determined for each pathogen-derived antigen. CliniMACS CCS runs yielded purity grades (54.5 ± 31.9% viable IFN-γ^+^ T cells) comparable to those reported in published studies [3,8].

The potency of antigen-specific IFN-γ^+^ T cells enriched by CliniMACS CCS and MiniMACS CSA were studied by several groups for various specificities (CMV, EBV, ADV, *Aspergillus*) [6,22,28-33]. These studies described the secretion of IFN-γ as suitable marker to determine the potency of the final TCF. In a previous study we were able to demonstrate that the isolation and enrichment did not alter the viability and antiviral T-cell function, observed by T-cell’s degranulation capacity, proliferation and secretion of the effector molecules IFN-γ, TNF-α and granzyme B after *in vitro* expansion [28]. However, preclinical studies with CliniMACS CCS-enriched T cells have demonstrated a significant lower alloreactivity after co-incubation with allogeneic APCs compared to unselected T cells. The loss of alloreactivity evidenced a high specificity of the generated virus-specific T-cell product [3,22,31-34]. In addition, the adoptive transfer of partially HLA-mismatched virus-specific cytotoxic T cells was shown to not induce *de novo* GvHD in the recipient the despite recognition of recipient HLA molecules [26].

For transplantation with unmanipulated CD34^+^ cells as well as DLIs a starting dose of <2.5 × 10^4^ CD3^+^ T cells/kg is recommended in a haploidentical setting and <10^5^ CD3^+^ T cells/kg in a HLA-matched setting to reduce the risk of inducing or enhancing GvHD [35]. In the post-transplant setting to test efficacy and safety the same total CD3^+^ T-cell safety limits will most likely apply to the adoptive T-cell transfer.

In the study of Peggs *et al.,* CMV-specific T cells isolated by CliniMACS CCS were used for adoptive transfer with a target T-cell dose of 1 × 10^4^ CD3^+^ T cells/kg of recipient body weight, a mean number of 2840 CMV-specific CD4^+^ T cells/kg body weight and 630 CMV-specific CD8^+^ T cells/kg body weight [8]. Icheva *et al.* 2012 isolated EBV EBNA1-specific T cells by CliniMACS CCS and used a mean number of 4.2x10^3^ CD3^+^ T cells/kg of recipient’s body weight with a mean number of 3613 EBV-specific CD4^+^ T cells/kg and 500 EBV-specific CD8^+^ T cells/kg for adoptive transfer [6]. In both studies, comparably low numbers of adoptively transferred antiviral CD3^+^ T cells were sufficient for the life-saving treatment.

In addition, we analysed the contribution of viable leukocyte subsets (CD3^+^CD56^+^ NKT cells, CD14^+^ monocytes, CD33^+^ granulocytes, CD19^+^ B cells, and CD3^−^CD56^+^ NK cells in the leukapheresis and the final TCF (Figure 6). It is likely that these contaminants were specifically captured during the CliniMACS CCS enrichment processes because of their ability to secret IFN-γ. Nevertheless, due to the *in vitro* antigen restimulation the total number of each analysed leukocyte subset is reduced in the final TCF (Figure 6). To gain more insight into the role of contaminating cell subsets in the TCF, the type and number of contaminants will be further investigated.

The enrichment of IFN-γ-positive T cells also resulted in (1) the enrichment of dead cells causing a comparably low viability (CliniMACS: 57.4 ± 5.62%) and (2) higher percentages of IFN-γ^+^ T cells in the WF than expected. Overall, little is known about the impact of dead adoptively transferred leukocytes. Despite the fact that the three validation runs performed complied with the defined acceptance criteria (Additional file 1: Table S1), improvement of the T-cell product’s viability is of great importance to provide sufficient numbers of functional active antiviral T cells. The use of the fully automated CliniMACS Prodigy (Miltenyi Biotec) might overcome these problems by e.g. shortening the process time to approximately 12 hours. Comparing the performance of the Prodigy with the CliniMACS CCS procedure will yield information on the viability, purity, and recovery of the enriched T-cell products.

Some circumstances might prevent the infusion of the antiviral TCF immediately after manufacture, including: (1) long-range logistics or (2) treatment plan-related issues, i.e., delayed infusion because of immediate life-saving procedures or repeated dosage schemes. Therefore we investigated stability of the TCF up to a period of 72 h and could clearly show stability of IFN-γ-positive T cells. Aliquots and reference samples of the three products were cryopreserved and stored in the gas phase above liquid nitrogen at < −160°C for subsequent stability testing and the validation of the cryopreservation process.

### Role of antigenic stimuli, T-cell subsets and T-cell numbers

The CMVpp65 peptide pool consists of 15-mer peptides of overlapping sequence spanning the whole viral target protein and enables the simultaneous induction of specific CD4^+^ and CD8^+^ T cells, irrespective of the HLA type [23,25,36,37]. As expected a higher HLA class I-restricted antigen-specific CD8^+^ T-cell response than for CD4^+^ T cells was observed.

It is not really clear yet what is the best ratio of antiviral CD4^+^ and CD8^+^ T cell to improve the efficacy of this procedure and clinical outcome. Feuchtinger and colleagues have analysed the number of naive, central memory, and effector memory T cells among IFN-γ-secreting CMVpp65-specific T cells in the TCF obtained via CliniMACS CCS enrichment [3]. They successfully identified that the largest subpopulation in the T-cell products is formed by T cells of late effector stages. This subset was detectable over six months after T-cell transfer and seems to be responsible for the long-lasting CMVpp65-specific T-cell immunity in transplant recipients. Moreover, many groups have shown that CD62L^+^ memory T cells may represent a suitable donor T-cell subpopulation for enhancing immune reconstitution and providing long-lasting immunity without increasing the risk of GvHD [3,38-40]. For this reason, it is of great importance to monitor the specific cellular immunity in patients before and after adoptive T-cell transfer in relation to the phenotype and quantities of transferred specific CD4^+^ and CD8^+^ T cells.

## Conclusions

Treatment of high-risk patients requires the efficient and rapid manufacture of virus-specific T cells without long-term *ex vivo* stimulation, while still maintaining antiviral CD8^+^ and CD4^+^ T cells including the rapid recruitment of specific seropositive T-cell donors with at least 3/6 HLA-matches to the patient. The manufacture of antiviral T cells using the CliniMACS CCS was validated at Hannover Medical School. By using the immunodominant CMVpp65 overlapping peptide pool during three independent CliniMACS CCS validation runs, a three-step protocol with standard criteria for donor selection and the rapid manufacturing of clinical-grade T cells was successfully designed to covering the entire procedure of antiviral T-cell generation for clinical applications. The accelerated selection and recruitment of third-party T-cell donors with adequate frequencies of virus-specific memory T cells from the *alloCELL* registry in the first two steps of the protocol will be feasible in less than three days at best, while clinical-grade T cells can be separated in less than 36 hours, thus allowing a short-term initiation of adoptive immunotherapy.

We separated the quality control into QC release criteria and additional report only analyses for our scientific “add on” (advanced characteristic) program. For this reason a product release document, which provides the release relevant product parameters: viability, total volume of the preparation, sterility, purity, percentage and cell numbers of IFN-γ^+^ as well as contaminating IFN-γ^−^ T cells was established. T-cell dose for adoptive transfer will be calculated regarding to the individual clinical requirements (e.g. differences between haploidentical and HLA-matched application).

The use of the IFN-γ-based CliniMACS CCS for specific T-cell enrichment offers a great flexibility by using any possible antigen of interest and results in products that are sufficiently uniform with regards to T-cell purity and in the depletion of unspecific IFN-γ^−^ T cells. The enrichment of virus-specific CD4^+^ and CD8^+^ T cells seems to be of great relevance for viral clearance and the long-lasting control of viral infections in a post-transplant setting. In addition, the established T-cell manufacturing protocol can be easily adapted to enrich T cells restricted against other pathogens such as EBV, ADV, HHV6 and *Aspergillus* and can be extended to melanoma (Melan-1/Mart-1) and tumor antigens (WT1).

Adoptive immunotherapy has become a valuable clinical extension to existing treatments. In the future it will be important to routinely monitor the patient’s cell-mediated immunity to identify high-risk patients who need adoptive T-cell transfer as a prophylactic treatment to improve their clinical outcome. Harmonization of risk-based and product release acceptance criteria between different GMP facilities will be helpful to identify the most relevant once. Increasing the availability of clinical safety and efficacy data for adoptive immunotherapy will help to define the clinical requirements (e.g., patient condition, T-cell frequency, optimal time point of transfer, viability and purity of the antiviral T-cell product) for an effective and safe therapeutic approach.

## Additional files

Additional file 1: Table S1. Overview of the acceptance criteria defined for the final T-cell product. Additional file 2: Table S2. Overview of antibody panels used in the flow cytometric analysis. Additional file 3: Table S3. Differentiation between CD8^+^ and CD4^+^ T cells in the T-cell fraction (TCF) by specific T-cell staining without (QCP-A) and with (QCP-C) anti-CD4 antibody.
