# Supplementary material for: Rapid generation of clinical-grade antiviral T cells: selection of suitable T-cell donors and GMP-compliant manufacturing of antiviral T cells
Source: J Transl Med. 2014 Dec 16;12:336. doi: 10.1186/s12967-014-0336-5 (PMC4335407; doi:10.1186/s12967-014-0336-5)
Supplement: Additional file 1: Table S1. — Overview of the acceptance criteria defined for the final T-cell product. [file 12967_2014_336_MOESM1_ESM.doc]

**Table S1. Overview of the acceptance criteria defined for the final** T-cell product.

| **acceptance criteria** | **leukapheresis** | **OF** | **TCF** |
| --- | --- | --- | --- |
| **viability** | ≥ 90% | ≥ 70% | ≥ 20% (“report only”) |
| **TNC** | ≥ 3x10^9^ | ≥ 5x10^8^ | determined |
| **CD3^+^ T cells** | ≥ 10% | determined | determined |
| **viable IFN-γ^+^ T cells** | ≥ 0.03% of total CD3^+^ T cells | determined | > 1x10^4^ |
| **viable IFN-γ^-^ T cells** | determined | determined | ≤ 2x10^7^ |
| **microbial control** | negative results | / | negative results |

In addition to a controlled GMP-compliant environment for the manufacture and testing, acceptance criteria were specifically defined for the starting fraction before (Leukapheresis) and after (OF, original fraction) restimulation with the CMVpp65 peptide pool and for the CliniMACS CCS-enriched T-cell fraction (TCF). TNC = total nuclear cells.
