# Supplementary material for: Rapid generation of clinical-grade antiviral T cells: selection of suitable T-cell donors and GMP-compliant manufacturing of antiviral T cells
Source: J Transl Med. 2014 Dec 16;12:336. doi: 10.1186/s12967-014-0336-5 (PMC4335407; doi:10.1186/s12967-014-0336-5)
Supplement: Additional file 2: Table S2. — Overview of antibody panels used in the flow cytometric analysis. [file 12967_2014_336_MOESM2_ESM.doc]

**Table S2**. Overview of antibody panels used in the flow cytometric analysis.

| **QCP-A** | **QCP-A^-^** | **QCP-B** | **QCP_C** |
| --- | --- | --- | --- |
|  | (FMO) |  |  |
| CD45-APC-Cy7 | CD45-APC-Cy7 | CD56-PE-Cy7 | CD45-APC-Cy7 |
| CD3-FITC | CD3-FITC | CD3-FITC | CD3-FITC |
| CD8-APC | CD8-APC | CD33-APC | CD8-PE-Cy7 |
| CD56-PE-CY7 | CD56-PE-CY7 | CD14-APC-H7 | CD4-APC |
| IFN-γ-PE | / | CD19-PE | IFN-γ-PE |
| 7AAD | 7AAD | 7AAD | 7AAD |

Quality and in-process control of the CliniMACS CCS fractions were performed by flow cytometry using three different staining panels (quality control panel A (QCP-A, composition of IFN-γ^+^ T cells), quality control panel B (QCP-B, composition of leukocyte subsets), and quality control panel C (QCP-C, differentiation between CD4^+^ and CD8^+^ T cells). As a control the QCP-A^-^ was used as fluorescence minus one (FMO) for IFN-γ. Fractions obtained during MiniMACS CSA were analysed by QCP-A^-^.
