# Supplementary material for: Rapid generation of clinical-grade antiviral T cells: selection of suitable T-cell donors and GMP-compliant manufacturing of antiviral T cells
Source: J Transl Med. 2014 Dec 16;12:336. doi: 10.1186/s12967-014-0336-5 (PMC4335407; doi:10.1186/s12967-014-0336-5)
Supplement: Additional file 3: Table S3. — Differentiation between CD8+ and CD4+ T cells in the T-cell fraction (TCF) by specific T-cell staining without (QCP-A) and with (QCP-C) anti-CD4 antibody. [file 12967_2014_336_MOESM3_ESM.doc]

**Table S3. Differentiation between CD8^+^ and CD4^+^ T cells in the T-cell fraction (TCF) by specific T-cell staining without (QCP-A) and with (QCP-C) anti-CD4 antibody.**

| **validation run** | **panel** | **viable CD3^+^ T cells [7AAD^-^/CD45^+^]** | **viable CD4^+^ T cells [CD3^+^]** | **viable CD8^+^ T cells [CD3^+^]** |
| --- | --- | --- | --- | --- |
|  |  | [%] | [%] | [%] |
| 1. run | QCP-A | 48.69 | 23.96 | 56.43 |
|  | QCP-C | 45.93 | 24.95 | 56.74 |
| 2. run | QCP-A | 64.23 | 25.48 | 13.49 |
|  | QCP-C | 63.08 | 25.84 | 13.53 |
| 3. run | QCP-A | 96.25 | 55.91 | 7.23 |
|  | QCP-C | 95.26 | 52.25 | 7.31 |
| **selected donor** | **used antibodies** | **Difference [%]** | **Difference [%]** | **Difference [%]** |
|  |  | [viable CD3^+^ T cells] | [viable CD4^+^ T cells] | [viable CD8^+^ T cells] |
| donor 1. run | CD8 vs CD8 + CD4 | **2.76** | **-0.99** | **-0.31** |
| donor 2. run | CD8 vs CD8 + CD4 | **1.15** | **-0.36** | **-0.04** |
| donor 3. run | CD8 vs CD8 + CD4 | **0.99** | **3.66** | **-0.08** |
| **selected donor** | **used antibodies** | **Standard Deviation [%]** | **Standard Deviation [%]** | **Standard Deviation [%]** |
|  |  | [viable CD3^+^ T cells] | [viable CD4^+^ T cells] | [viable CD8^+^ T cells] |
| donor 1. run | CD8 vs CD8 + CD4 | **1.95** | **0.70** | **0.22** |
| donor 2. run | CD8 vs CD8 + CD4 | **0.81** | **0.25** | **0.03** |
| donor 3. run | CD8 vs CD8 + CD4 | **0.70** | **2.59** | **0.06** |

Specific T-cell staining was performed for all CliniMACS CCS fractions by using two different staining panels without (QCP-A) and with (QCP-C) the anti-CD4 monoclonal antibody (mAB). The results for the representative analysis of the cells from the T-cell fraction (TCF) are shown. For phenotypic analysis with the anti-CD4 mAB, (A) CD4^+^ T cells [%] were gated on viable CD3^+^ T lymphocytes. The gating strategy for T-cell staining without anti-CD4 mAB (B) was specified by gating CD4^+^ T cells [%] on viable CD3^+^CD8^-^ T lymphocytes. Statistical analysis is displayed as the difference [%] and standard deviation [%].
